# The Change in Trait Resilience Predicts the Alleviation of Transdiagnostic Depressive Symptoms in Outpatient Adolescents: The Mediating Role of the Change in Psychological Inflexibility/Experiential Avoidance

**DOI:** 10.1002/cpp.70268

**Published:** 2026-04-09

**Authors:** Katriina M. Sarnola, Hanna Välitalo, Siiri‐Liisi Kraav, Sebastian Therman, Karoliina Kurkinen, Sami‐Matti Ruuska, Virve Kekkonen, Petri Kivimäki, Soili M. Lehto, Tommi Tolmunen

**Affiliations:** ^1^ Department of Psychiatry Kuopio University Hospital Kuopio Finland; ^2^ Institute of Clinical Medicine University of Eastern Finland Kuopio Finland; ^3^ Department of Social Sciences, University of Eastern Finland, Kuopio Campus Finland; ^4^ Mental Health Unit, Finnish Institute for Health and Welfare Helsinki Finland; ^5^ Institute of Clinical Medicine University of Oslo Oslo Norway; ^6^ R&D Department, Division of Mental Health Services Akershus University Hospital Lørenskog Norway; ^7^ Department of Psychiatry University of Helsinki Helsinki Finland

**Keywords:** adolescent, depressive symptoms, experiential avoidance, psychological inflexibility, trait resilience

## Abstract

This study examined how the change in trait resilience, an increasing tendency to effectively cope with challenges and flexibly adapt to stress, affected the change in transdiagnostic depressive symptoms over 6 months of clinical follow‐up among outpatient adolescents, and the role of psychological inflexibility/experiential avoidance (PI/EA), characterised by rigid and avoidant emotion regulation, in this association. Trait resilience was measured with the Brief Resilience Scale, PI/EA with the Acceptance and Action Questionnaire‐II and depressive symptoms with the Beck Depression Inventory‐IA. Questionnaires were completed at baseline and on 6‐month follow‐up by 337 study participants. Paired sample *t*‐tests were conducted to measure the change in the scale scores. Linear regression was used to assess the impact of the change in trait resilience on the change in depressive symptoms. Mediation analysis was performed to investigate the role of the change in PI/EA in the association between the change in trait resilience and the change in depressive symptoms. The change in trait resilience predicted the change in depressive symptoms, which was mediated by the change in PI/EA. Assessment of trait resilience and interventions focused on reducing PI/EA are suggested for the alleviation of transdiagnostic depressive symptoms in outpatient adolescents.

## Summary

- Outpatient adolescents answered the study questionnaires at study baseline and at 6 months follow‐up in a naturalistic clinical study setting.
- The change in trait resilience, i.e., the increasing ability to bounce back and recover from adversities, predicts improvement in transdiagnostic depressive symptoms.
- Psychological inflexibility/experiential avoidance (PI/EA) seems to be an important malleable emotion regulation strategy to be evaluated in the context of trait resilience.
- The decline in PI/EA during the naturalistic clinical follow‐up may be a proxy of resilience.

## 1 Introduction

During recent years, resilience has become an important research topic in clinical psychology. This has possibly been driven by increased interest in early intervention strategies and the prevention of mental illness (Kalisch et al. 2015). Moreover, the growing interest in positive aspects of mental health, regardless of psychological difficulties, might also have influenced this paradigm shift (Kalisch et al. 2017). Resilience may protect against several psychiatric conditions throughout the life span (Masten et al. 2021; McLaughlin et al. 2020; Stainton et al. 2018). Therefore, modifiable factors that influence resilience or its impact are of particular interest (Ong et al. 2009; Rutter 2006). Especially during adolescence, when most psychiatric problems arise (McGrath et al. 2023), it is important to gain a better understanding of the effects of resilience on mental health.

Depression is a major health concern among adolescents and has long‐term consequences for both the affected individuals and society (Breslau et al. 2008; Whiteford et al. 2013). It is therefore crucial to identify factors that help to prevent the development or worsening of depressive symptoms in adolescents (Askeland et al. 2020). On the population level, depressive symptoms increase between childhood and adolescence, especially among girls (Thapar et al. 2012), and exposure to psychological stress is one of the strongest risk factors for depression (Askeland et al. 2020; Garber 2006). During adolescence, depressive symptoms often occur in conjunction with other mental health problems, and diagnostic definitions of the adolescent mental health state may vary, especially intrapersonally (Shah et al. 2020). Hence, it is reasonable to evaluate depressive symptoms transdiagnostically and dimensionally, not restricting to a specific psychiatric diagnosis (Shah et al. 2020). Moreover, depressive symptoms appear to be crucial and transdiagnostic in all psychiatric disorders, and comorbid depressive symptoms are associated with an increased suicide risk, poor prognosis and reduced quality of life (Nakajima et al. 2023).

Resilience predicts fewer depressive symptoms in adolescence (Chung et al. 2020; Hjemdal et al. 2007, 2011). In a clinical study group of adolescents with attention deficit disorder (ADHD), individual resilience characteristics were associated with less depression and anxiety, as well as better psychosocial functioning (Schei et al. 2018). Moreover, resilience may have an important role in recovery from mental illness, as it appears to improve the treatment response in patients with depressive disorder (Min et al. 2012), even after psychotherapeutic interventions in adults (Konradt et al. 2018) and to lower the overall psychiatric symptom load in adolescent psychiatric patients (Gårdvik et al. 2021). However, the exact mechanism through which resilience affects mental health remains unresolved.

Definitions of resilience vary widely, leading to diverse research approaches (Kalisch et al. 2017; Stainton et al. 2018). However, in all definitions, resilience is described as positive adaptation in the face of adversity (Mesman et al. 2021). Moreover, resilience has been defined as a trait that is relatively stable over time and across different types of adversity, helping individuals cope with and adjust to the challenges (J. Block and Kraemen 1996; Hu et al. 2015; Smith et al. 2008). Trait resilience is often characterised by toughness and perseverance, which are measured with self‐rated questionnaires (Campbell‐Sills and Stein M. 2007; Connor and Davidson 2003). The capacity to cope and adapt to changes, recover from stress and bounce back from setbacks is also a central feature of trait resilience (Smith et al. 2008). Trait resilience predicts depression and anxiety, and it offers a relatively stable prediction of an individual's mental health (Hu et al. 2015). Recently, however, it has been increasingly acknowledged that trait resilience might be only one aspect of the broader concept of resilience (Mondolin et al. 2024; Ungar and Theron 2020), and that it might also be sensitive to change (Cosco et al. 2017; Chen et al. 2025; Meule et al. 2024; Mondolin et al. 2024). Specifically, in the clinical population, trait resilience has been demonstrated to significantly improve during the inpatient treatment of depressed adults (Meule et al. 2024).

Stable, trait‐like resilience characteristics make resilient reactions to stressors possible by promoting individual coping mechanisms (Kalisch et al. 2017). For example, trait resilience can promote favourable interactions with the environment or enable one to utilise favourable emotion regulation in stressful situations (Kalisch et al. 2017). In this way, trait resilience may prevent stress‐related mental health problems and facilitate recovery from mental health challenges (Hu et al. 2015). In this study, we refer to resilience as a trait according to the measure that we used, the Brief Resilience Scale (BRS) (Smith et al. 2008). However, previous research regarding the protective and predictive role of the change in trait resilience in mental health outcomes is scarce. Therefore, we were interested in the change in trait resilience.

The adolescent resilience framework theory states that the assets of resilience may come from its internal or external resources, including personal competencies, such as self‐esteem, and the environment, such as social support (Fergus and Zimmerman 2005). Trait‐like resilience characteristics could be considered as internal resources of resilience and have been demonstrated to be connected to different emotion regulation strategies (Lazarus 1984; Li and Zheng 2025; San Román‐Mata et al. 2020). Trait resilience promotes adaptive coping and might be associated with positive emotion regulation, because coping through emotions has been observed to mediate the effect of resilience on mental well‐being in juveniles (Konaszewski et al. 2021). In contrast, maladaptive coping strategies, such as disengagement coping (i.e., efforts to orient away from the source of stress or one's emotions, including avoidance or denial) and emotional suppression (i.e., efforts to dampen internal or external experiences and/or expressions of emotion) have predicted higher levels of psychopathological symptoms (Compas et al. 2017). Strategies focused on denial/avoidance or distraction and minimisation are negatively associated with resilience (Campbell‐Sills et al. 2006; Konazewski et al. 2021). Individuals with lower levels of trait resilience tend to utilise expressive suppression as a way of regulating emotions (Alismail and Almulla 2023). In the study by Ma et al. (2022), Chinese adolescents with low trait resilience were more prone to negative feelings such as anxiety, depression and stress when facing adversities, and they relied on avoidant coping strategies such as avoidance or denial. These avoidant means of emotion regulation and coping are typical in experiential avoidance (EA), which is a central part of psychological inflexibility (PI) according to the Acceptance and Commitment Therapy (ACT) theory (S. C. Hayes et al. 1996).

EA is described as an unwillingness to stay in contact with unwanted inner experiences, which refers to efforts to control or avoid unpleasant emotions, upsetting memories, troubling thoughts or physical sensations (S. C. Hayes et al. 1996). PI, on the other hand, is a maladaptive coping strategy in which a person's reactions to their thoughts and feelings dominate their behaviour, despite their values (Bond et al. 2011; L. Hayes et al. 2011; Levin et al. 2014). It is a key concept of ACT, in which behavioural models of mindfulness and acceptance are employed to increase psychological flexibility and thereby alleviate psychological distress (S. C. Hayes et al. 2006). In earlier studies, both EA and PI have been used as measures of avoidant coping styles and maladaptive emotion regulation strategies. In one study, a resilient personality (J. H. Block and Block 1980; J. Block and Kraemen 1996) was predictive of lower levels of depressive symptoms over time through its beneficial influence on lower avoidant coping and PI (Elliott et al. 2015).

PI/EA functions as a transdiagnostic process in a range of psychiatric disorders, including depressive and anxiety disorders, substance use disorders and eating disorders, as well as comorbidity across disorders, which means that all these conditions may be associated with psychologically inflexible adjustment (Levin et al. 2014; Mervin et al. 2010; Venta et al. 2012). PI/EA is a significant predictor of various forms of psychopathology, including depression and anxiety (Wang et al. 2024). In two cross‐sectional adolescent studies, PI/EA was linked to depression and anxiety at both diagnostic and symptom levels (Chawla and Ostafin 2007; Venta et al. 2012). Moreover, PI/EA was elevated in female adolescents who had not yet developed major depressive disorder (MDD; Mellick et al. 2017). PI/EA may predict chronic MDD and/or generalised anxiety disorder (GAD), and it is more consistently elevated in adolescents with persistent MDD and GAD symptoms (Mellick et al. 2019). Furthermore, PI has proven to be an important factor in the maintenance and prediction of depression in adolescence (Chawla and Ostafin 2007; Mellick et al. 2019; Venta et al. 2012), and the alleviation of depressive symptoms may result from a decline in PI/EA (Berking et al. 2009).

While trait resilience promotes individual coping mechanisms (Kalisch et al. 2017) and is associated with positive coping and emotion regulation, it might be negatively associated with emotion dysregulation and maladaptive coping strategies, such as PI/EA. It is also established that resilience predicts positive outcomes in mental health problems, while PI/EA predicts mental health problems and their persistence. Understanding the interrelationships between trait resilience, PI/EA and depressive symptoms may help in the promotion of adolescent mental health, because PI/EA might serve as a transdiagnostic treatment target for various forms of psychopathology in adolescents (Keulen et al. 2023; Mellick et al. 2019; Wang et al. 2024). In fact, it is still not known whether a decline in PI/EA could act as a resilience mechanism to promote mental health by linking trait resilience to reduced depressive symptoms. Moreover, it is also not known how the change in trait resilience in adolescents during a naturalistic clinical follow‐up is associated with the improvement in depressive symptoms. Hence, in this naturalistic, longitudinal clinical study design, we hypothesised that the change in trait resilience predicts the alleviation of transdiagnostic depressive symptoms and is associated with a greater decline in PI/EA, which in turn would lead to lower levels of depressive symptoms during a 6‐month follow‐up of outpatient adolescents. We exploratively conducted a mediation analysis to assess the effect of the decline in PI/EA on the association between the change in trait resilience and the change in depressive symptoms over 6 months of naturalistic follow‐up in outpatient adolescents.

## 2 Methods

### 2.1 Sample

This study was a secondary data analysis of the REAL‐SMART project (“Recognition and early intervention for alcohol and substance abuse in adolescence and systemic metabolic alterations related to different psychiatric disease categories in adolescent outpatients”), the primary aim of which was to evaluate various psychiatric diagnostic profiles and predict the sequelae of the psychiatric state in adolescence with different methods, such as metabolomics. In the present study, all 14‐ to 20‐year‐old patients (*n* = 2817) who were admitted to the adolescent psychiatric outpatient clinic of Kuopio University Hospital between June 2017 and May 2025 were approached. The only exclusion criterion was the inability to understand the Finnish‐language study questionnaires. At baseline, consenting participants were interviewed and asked to complete self‐rated questionnaires on a laptop computer. The interview included the Structured Clinical Interview for DSM‐IV disorders (SCID‐IV; First et al. 2017), which was performed by a trained psychiatric nurse. The participants were interviewed again on 6‐month follow‐up and completed an identical set of self‐rated questionnaires on an Android tablet computer using OpenODK Collect (Hartung et al. 2010). Answering the study questionnaires took approximately 2 h per occasion. There was no compensation for participation in the study. All participants provided written informed consent, and the consent was also approved by the caregivers of participants who were under 15 years old at the study baseline, as mandated by national guidelines. The parents of minors aged 15 or older were also informed of the study.

During the study, there were some periods during which no study nurses were available to perform the follow‐up assessments, which naturally increased the dropout rate between the baseline and the follow‐up. The COVID‐19 pandemic also affected the dropout rate because of lockdown periods during the study. The mental health status and the treatment received by the study participants might have influenced the dropout rate, because some participants discontinued psychiatric treatment in the outpatient clinic. We restricted the analyses to examining data from those who had completed all measurements at baseline and on 6‐month follow‐up. Of the baseline cohort, 2003 refused to participate, leaving a total of 814 participants, of whom 337 had completed all the study questionnaires at both time points (at baseline and on 6‐month follow‐up) by May 2025.

Of the participants in the final dataset, 73 were male and 264 were female, and their mean age was 16.5 (SD 1.6, range 13–19) years. During the follow‐up period, all participants received ongoing clinician‐based case management and relevant social, psychological and medical treatment as part of standard care. This might have involved contact with a psychiatrist, psychologist, therapist, or social support worker and hospitalisation for patients whose needs exceeded the capacity of the outpatient clinical services. The study conformed to the standards set by the 7th revision of the Declaration of Helsinki (World Medical Association 2013). The study protocol was approved by the Research Ethics Committee of Kuopio University Hospital.

### 2.2 Questionnaires

#### 2.2.1 Depression

Depressive symptoms were assessed with the Beck Depression Inventory‐IA (BDI‐IA; Beck et al. 1996). The BDI‐IA is a 21‐item measure of current depressive symptoms, which is widely used globally and is known to have strong psychometric properties (Beck et al. 1996; Steer et al. 1998). Items are scored from 0 to 3 and are summed for a total score of 0 to 63. Cronbach's alpha for the BDI was 0.93 at baseline and 0.95 on follow‐up.

#### 2.2.2 PI

The Acceptance and Action Questionnaire‐II (AAQ‐II, Bond et al. 2011) was used to assess PI/EA as a self‐report measure in our study. The AAQ‐II consists of seven items, with responses on a 7‐point rating scale (1 = never true, 7 = always true). Examples of sample items include “Emotions cause problems in my life” and “Worries get in the way of my success”. Increased AAQ‐II scores are associated with measures of depressive symptoms, anxiety, stress and greater overall psychological distress (Bond et al. 2011). However, the AAQ‐II appears to address a separate construct, which may predict mental health problems. Items were scored so that higher scores indicated greater PI. The AAQ‐II has previously been found to have good internal consistency and test–retest reliability (Bond et al. 2011). Cronbach's alpha was 0.90 at baseline and 0.93 on follow‐up.

#### 2.2.3 Resilience

The BRS (Smith et al. 2008) was used to assess self‐reported trait resilience. The BRS has six items, including “I tend to bounce back quickly after hard times” and “I have a hard time making it through stressful events”. High BRS scores appear to be uniquely related to health, and especially to reducing anxiety, depression, negative affect and physical symptoms. The BRS has earlier demonstrated good internal consistency, convergent validity and discriminant validity (Smith et al. 2008). Cronbach's alpha was 0.89 at baseline and 0.91 on follow‐up.

### 2.3 Statistical Analysis

Descriptive statistics for all variables and Pearson correlations between variables were calculated using SPSS version 27.0.1. To analyse the change in AAQ‐II, BDI‐IA and BRS scores, we used paired‐sample *t*‐tests. We tested the effect of the change in trait resilience on the change in depressive symptoms with linear regression analysis, with the change in trait resilience, age, gender, baseline depressive symptoms and baseline trait resilience as predictors/covariates, and the change in depressive symptoms as an outcome measure, and additionally examined the mediating effect of the change in PI/EA. *P*‐values under 0.05 were considered statistically significant. Gender, age, baseline resilience and baseline depressive symptoms were controlled in the linear regression analysis and mediation analysis. In the mediation analysis, PI/EA at baseline was also considered as a covariate. Simple mediation analysis was performed with the PROCESS macro v.3.5 for SPSS (A. F. Hayes 2018). The PROCESS macro is based on ordinary least squares (OLS) regression analysis and takes into account the assumption of causality of the studied variables, the validity of the measures, and the independence of the observations. Linearity, multicollinearity, homoscedasticity and the outliers were tested separately to confirm that our model satisfied the assumptions of OLS (Supplementary Material 2). The standard number of bootstrap samples (5000) was used. If the upper and lower bounds of 95% confidence intervals did not contain zero, the indirect effect was considered significant. All of the statistical analyses were performed with SPSS version 27.0.1.

## 3 Results

### 3.1 Correlations Between Variables

Resilience measured at baseline displayed a strong negative correlation with PI/EA at baseline and a moderate negative correlation on 6‐month follow‐up. Baseline resilience was also strongly negatively correlated with depression at baseline and moderately with depression on 6‐month follow‐up. Greater resilience at baseline was associated with a greater improvement in depressive symptoms, i.e., a decline in depressive symptoms on average. There was also a strong positive relationship between PI/EA and depressive symptoms both at baseline and on follow‐up. Age was weakly associated with PI/EA at baseline and the change in depression and PI/EA. The change in resilience correlated moderately with the change in depressive symptoms and strongly with the change in PI/EA (Table 1).

**TABLE 1:** Pearson correlation coefficients for the relationships between the continuous variables used in the linear regression model (*n* = 337).

	BDI	BDI	BDI	AAQ‐II	AAQ‐II	AAQ‐II	BRS	BRS	BRS
	BL	F/U	Δ	BL	F/U	Δ	BL	F/U	Δ
Age	0.08	−0.06	−0.1**	0.18**	0.04	−0.15**	−0.12*	−0.03	0.10
BDI BL		0.66***	−0.34***	0.64***	0.53***	−07	−0.54***	−0.50***	0.05
BDI F/U			0.47***	0.44***	0.70***	0.37***	−0.37***	−0.60***	−0.29***
BDI Δ				−0.20***	0.25***	0.54 ***	0.18**	−0.16**	−0.42***
AAQ‐II BL					0.65***	−0.32***	−0.68***	−0.55***	0.15**
AAQ‐II F/U						0.51***	−0.47***	−0.70***	−0.28***
AAQ‐II Δ							0.17**	−0.24***	−0.51***
BRS BL								0.67***	−0.40***
BRS F/U									0.42***

Abbreviations: Δ, change; AAQ‐II, Acceptance and Action Questionnaire II; BDI, Beck Depression Inventory; BL, baseline; BRS, Brief Resilience Scale; F/U, follow‐up.

* *p* < 0.05.

** *p* < 0.01.

*** *p* < 0.001.

### 3.2 Changes in Outcome Measures

BDI scores were lower on 6‐month follow‐up, as were AAQ‐II scores (Table 2). In contrast, there was no statistically significant change in BRS scores (Table 2).

**TABLE 2:** BDI, AAQ‐II and BRS scale scores for the whole sample at baseline and on 6‐month follow‐up and the change over time (*n* = 337).

	Mean (SD) at baseline	Mean (SD) on 6‐month follow‐up	Mean (SD) score change	Test value^a^	*p*	Effect size of change (Cohen's *d*)
BDI	22.97 (12.67)	19.83 (13.48)	−3.15 (10.75)	5.37	<0.001	0.29
AAQ‐II	25.80 (10.03)	23.96 (11.09)	−1.84 (8.89)	3.80	<0.001	0.21
BRS	2.75 (0.83)	2.80 (0.84)	0.05 (0.67)	−1.33	0.37	−0.07

^a^ Paired samples *t*‐test.

### 3.3 Direct Effect of Resilience on Depressive Symptoms

In the linear regression, the change in trait resilience predicted the change in depressive symptoms, including when baseline depression, baseline resilience, age and gender were entered as covariates (Table 3). Multicollinearity was tested using variance inflation factors (VIF), which indicated very little variance inflation.

**TABLE 3:** Results of a linear regression model predicting the change in BDI scores (*n* = 337).

	*β*	*t*	*p*	*R*‐square	Adjusted *R*‐square	VIF
All variables			<0.001	0.34	0.33	
Age	−0.10	−2.21	0.03			1.03
Gender	0.07	1.39	0.17			1.14
BRS baseline	−0.27	−4.53	<0.001			1.82
ΔBRS	−0.50	−9.92	<0.001			1.26
BDI baseline	−0.48	−8.77	<0.001			1.52

Abbreviations: Δ, change; AAQ‐II, Acceptance and Action Questionnaire II; BDI, Beck Depression Inventory; BL, baseline; BRS, Brief Resilience Scale; F/U, follow‐up.

### 3.4 Mediating Effect of PI/EA

**FIGURE 1:**
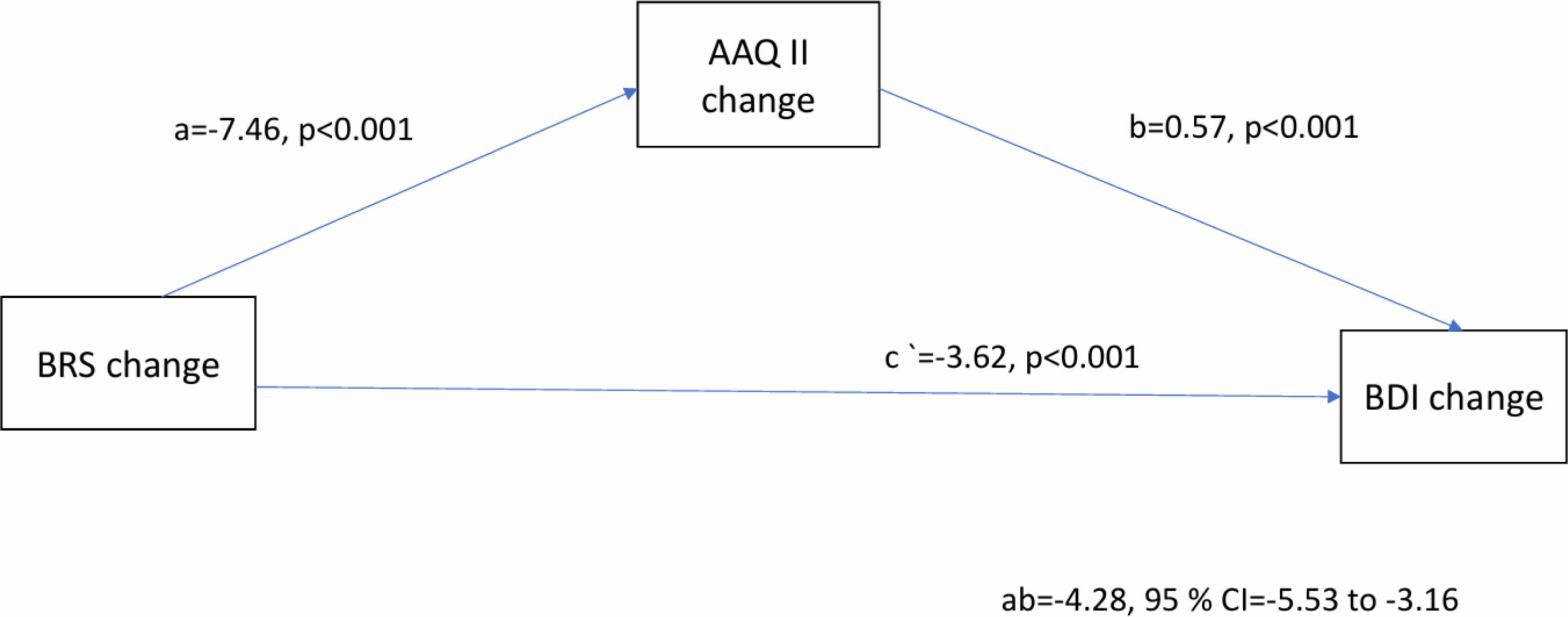
Mediation model adjusted for age, gender, baseline BDI, baseline BRS and baseline AAQ‐II, the coefficients of which are not shown for the sake of simplicity and clarity (*n* = 337). Abbreviations: AAQ‐II, Acceptance and Action Questionnaire II; BDI, Beck Depression Inventory; BRS, Brief Resilience Scale.

In the simple mediation analysis, the change in trait resilience indirectly affected the level of change in depressive symptoms through the change in PI/EA (Table 4; Figure 1). The change in trait resilience was associated with a greater change in PI/EA (*a* = −7.46), which in turn resulted in a greater change in depression between baseline and follow‐up (*b* = 0.57). The 95% bootstrap confidence interval for the indirect effect (*ab* = −4.28) was entirely under zero (−5.53 to −3.16). The change in trait resilience also directly influenced the change in depression (c' = −3.62, *p* < 0.001). The mediation analysis was adjusted for age, gender, baseline depressive symptoms, baseline trait resilience and baseline PI/EA.

**TABLE 4:** Regression coefficients, standard errors (SE) and model summary information for the simple mediation model assessing the effect of BRS change (X) on BDI change (Y) through changes in AAQ‐II (M). The results are adjusted for five possible confounders: age at baseline (U_1_), gender (U_2_), baseline BDI (U_3_)_,_ baseline BRS (U_4_) and baseline AAQ‐II (U_5_) (*n* = 337).

Antecedent	Consequent							
	Mediator (M) (AAQ‐II change)				Dependent (Y) (BDI change)			
		Coeff.	SE	*p*		Coeff.	SE	*p*
X (BRS change)	*a*	−7.46	0.63	<0.001	*c’*	−3.62	0.86	<0.001
M (AAQ‐II change)		—	—	—	*b*	0.57	0.06	<0.001
U_1_ Age	*a*_2_	−0.19	0.23	0.42	*c´*_2_	−0.54	0.27	0.04
U_2_ Gender	*a*_3_	1.99	0.97	0.04	*c´*_3_	0.58	1.12	0.60
U_3_ Baseline BDI	*a*_4_	0.07	0.04	0.08	*c´*_4_	−0.45	0.05	<0.001
U_4_ Baseline BRS	*a*_5_	−3.70	0.71	<0.001	*c´*_5_	−1.43	0.84	0.09
U_5_ Baseline AAQ‐II	*a*_6_	−0.48	0.06	<0.001	*c’*_6_	0.28	0.07	<0.001
Constant	*i*_*M*_	21.14	4.92	<0.001	*i*_*y*_	13.60	5.76	0.02
		*R* ^2^ = 0.41				*R* ^2^ = 0.48		
		*F*(330) = 38.21, *p* < 0.001				*F*(329) = 42.77, *p* < 0.001		
		*ab* = −4.28; Boot SE = 0.61; 95% CI (boot) = −5.53 to −3.16						

Abbreviations: AAQ‐II, Acceptance and Action Questionnaire II; BDI, Beck Depression Inventory; BRS, Brief Resilience Scale.

## 4 Discussion

To the best of our knowledge, this was the first study to investigate the longitudinal associations between the change in trait resilience, PI/EA and depressive symptoms in adolescents in a naturalistic clinical setting. We observed a negative correlation between trait resilience and PI/EA at baseline. Depressive symptoms also correlated negatively with trait resilience and positively with PI/EA. The change in trait resilience was associated with the alleviation of depressive symptoms. In mediation analysis, the change in trait resilience indirectly affected the level of change in depressive symptoms through the change in PI/EA after adjusting the model for age, gender, baseline depressive symptoms, baseline trait resilience and baseline PI/EA. There was also a direct and significant effect of the change in trait resilience on the change in depressive symptoms during the follow‐up.

Based on our findings, the change in trait resilience appears to predict the alleviation of transdiagnostic depressive symptoms. Similarly, in a clinical adolescent population, higher self‐reported resilience was linked with a lower severity of psychiatric symptoms after 3 years of follow‐up, regardless of the psychiatric diagnosis (Gårdvik et al. 2021). In another study, higher resilience scores predicted lower levels of depression, anxiety and obsessive‐compulsive symptoms in adolescents (Hjemdal et al. 2011). Moreover, in a naturalistic sample of inpatient adults with depression, higher trait resilience predicted larger decreases in depressive symptoms (Meule et al. 2024). However, previous results regarding the protective and predictive role of the change in trait resilience in mental health outcomes are scarce.

Overall, resilience also appears to associate with better mental health outcomes in adolescence. Wu et al. (2020) demonstrated that resilience predicted the 1‐year mental health status in a general population sample of undergraduate students, but resilience did not predict good mental health during the second year. The current study is in line with these findings, indicating that trait resilience may have protective qualities in connection with adolescent mental health and that the change in trait resilience significantly affects the improvement in transdiagnostic depressive symptoms. In our study, we used the BRS as the measure of resilience. The BRS was originally developed as a reliable and unitary construct to measure an individual's ability to bounce back or recover from stress (Smith et al. 2008), and it represents a factor solution that is related to resilience resources and health outcomes (Zhang et al. 2020).

In previous studies, psychiatric patient groups have displayed lower levels of resilience than general population controls (Pardeller et al. 2020; Seok et al. 2012). In contrast, a subgroup of patients with depression and/or anxiety disorders who were characterised by higher scores in spirituality and purpose in life questionnaires reported moderate or even high levels of resilience (Min et al. 2013). Therefore, a depressive state does not necessarily directly indicate low resilience, and resilience is not simply the absence of psychopathology (Pardeller et al. 2020; Waugh and Koster 2015). Even though most of our study participants already suffered from a mental health condition, many appeared to be resilient enough for an improvement in their depressive symptoms to occur. Moreover, in some cases, trait resilience even improved during the follow‐up, which significantly impacted the alleviation of depressive symptoms, also in line with the earlier findings (Chen et al. 2025; Meule et al. 2024). According to our results, trait resilience is sensitive to some change which can, in turn, lead to a positive outcome regarding adolescent mental health. An increasing ability to adapt to adversities appears to have a crucial role not only in preventing depression but also in alleviating it.

Consistently with previous studies, resilience and PI/EA negatively correlated with each other (Elliott et al. 2015; 2017; Meyer et al. 2019). The correlation between resilience and depressive symptoms was also negative, mirroring earlier findings (Chung et al. 2020; Elmore et al. 2020; Goldenson et al. 2021; Hu et al. 2015; Hjemdal et al. 2007, 2011; Miller and Chandler 2002; Nrugham et al. 2010; Poole et al. 2017; Shapero et al. 2019). Our results are also in line with previous findings indicating that higher levels of resilience associate with lower levels of depressive symptoms in adolescents (Chung et al. 2020; Elmore et al. 2020; Goldenson et al. 2021; Hjemdal et al. 2007, 2011). These findings suggest that resilience is negatively linked with mental ill health and positively with an improvement in mental health problems, such as lower depressive symptoms.

Our mediation analysis indicated that the effect of the change in trait resilience on the improvement in depressive symptoms was mediated by the change in PI/EA. However, the direct effect of the change in trait resilience on the change in depressive symptoms was also significant. Our findings support an earlier report of a resilient personality having a significant indirect effect on self‐reported good mental health through its beneficial effects on PI (Elliott et al. 2015). Maladaptive coping and emotion dysregulation strategies are associated with psychopathology in youth, and the habitual use of avoidance has been linked to depressive and anxiety symptoms (Schafer et al. 2017; Siu and Shek 2010). Similarly, PI/EA appears to underlie many psychopathological conditions in youth (Levin et al. 2014). Improvements in PI/EA are associated with better mental health, such as diminished depressive symptoms (Berking et al. 2009), which is also in line with our study findings. Accordingly, changes in trait resilience might improve depressive symptoms by enhancing emotion regulation strategies, thereby reducing avoidant emotion regulation (Li and Zheng 2025). In this sense, the change in trait resilience appears to have protective and adaptive qualities by promoting the reduction in PI/EA, which in turn leads to a reduction in depressive symptoms.

As prior research has demonstrated, PI/EA is a malleable emotion regulation strategy that can be modified using a range of psychosocial interventions. ACT, in particular, is designed to reduce PI/EA in order to improve the functioning of people experiencing a range of mental health challenges (S. C. Hayes et al. 2006; Walser et al. 2015). This integrated perspective considering the change in trait resilience, PI/EA and depressive symptoms advances our understanding of the psychological protective effects of trait resilience and the mechanisms underlying the improvement in transdiagnostic depressive symptoms.

A key strength of our study was the longitudinal study design, which allowed us to observe the associations between the change in trait resilience, PI/EA and depressive symptoms in a naturalistic clinical follow‐up. Moreover, the data were not divided based on the specific psychiatric diagnosis or more specified psychiatric symptoms in accordance with the emphasis of the PI/EA model, which refers to the dimensional conceptualisation of psychopathology and health (L. Hayes et al. 2011; Levin et al. 2014.) Because of the naturalistic approach, we did not standardise the studied associations according to the received treatment or its duration. However, these might have influenced the associations between trait resilience, PI/EA and changes in depressive symptoms and should be considered a limitation of our study. Moreover, we used a PROCESS‐based macro to examine mediation and acknowledge that other methods might outperform regression‐based mediation.

The study participants were ethnically homogeneous Caucasians, allowing us to interpret the findings only in this population. Because all the participants were outpatients of a university hospital, to which they were usually referred by primary practitioners, potential selection bias related to the participants may limit the generalisation of our findings. Thus, it remains unclear how well these results can be generalised to other populations, such as healthy adolescents, adults and various population subgroups. As our sample was a naturalistic clinical sample with depressive symptoms being the reasons for involvement, girls were overrepresented. Gender was used as a covariate in the multivariate and mediation analyses.

Due to the COVID‐19 pandemic, the study interviews were interrupted several times. During the study, there were periods during which no study nurses were available to perform follow‐up assessments. The COVID‐19 pandemic also affected the dropout rate because lockdown periods occurred during the study. Moreover, the mental health status and the treatment received by the study participants might have influenced the dropout rate because the sample included participants who were no longer receiving psychiatric treatment from the outpatient clinic.

Because the follow‐up time of the study was relatively short, the effect of resilience on depressive symptoms needs to be further investigated in longitudinal research settings with longer follow‐up times to determine the long‐term impact of the change in trait resilience on depressive symptoms. Furthermore, we did not have information on the inflexibility and trait resilience levels of the participants before developing mental health conditions. Self‐report measurement of trait resilience, depressive symptoms and PI/EA while the participants were suffering from mental health conditions may have tempered the reliability of the study results.

Moreover, the unequal gender distribution of the study sample, significant dropout rate and relatively low overall response rates may have caused potential selection bias and should be considered in interpreting the present findings. Due to the naturalistic clinical study design, we did not consider several detailed clinical characteristics or psychosocial determinants that might potentially influence PI/EA and the change in depressive symptoms, such as the specific psychiatric diagnosis, medical treatment or its duration or social support.

Our study demonstrated that the greater the improvement in trait resilience during the clinical follow‐up, the more likely the depressive symptoms were to be alleviated in the participating adolescents. This association was mediated by the change in PI/EA. There was also a significant direct effect of the improvement in trait resilience on the alleviation of depressive symptoms. This study additionally provided new insights into resilience‐related factors, indicating that the change in PI/EA, i.e., the decline in PI/EA, is a proxy for resilience that mediates the effect of the change in trait resilience on the improvement in depressive symptoms. We found the mediating effect of the change in PI/EA to significantly explain the association between the change in trait resilience and the change in depressive symptoms in this naturalistic outpatient sample. Even though resilience research has in the past few decades shifted from approaching and investigating the construct as a trait, trait‐like resilience characteristics appear to be associated with coping and emotion regulation strategies, which in turn may have a role in recovery from mental health problems.

Mental health specialists could evaluate the level of trait resilience in adolescents and help them to use their resilience with diverse strategies. Specifically, those at risk due to their lower levels of trait resilience are of special interest, because with targeted interventions and training, they might have the opportunity to utilise their resilience and its resources (Hu et al. 2015). According to our results, the change in trait resilience explains the variance in the change in PI/EA, which in turn explains the variance in the change in depressive symptoms. Trait resilience appears to be associated with emotion regulation strategies and coping. Hence, it is important to evaluate how adolescents relate to their thoughts and feelings, and to gain a better understanding of their PI/EA tendencies simultaneously with trait resilience. Cognitive therapies such as ACT, which focus on one's values, emotions, thoughts and appraisal styles and address PI/EA, might provide an important means to reduce PI/EA, which in turn might have an influence on the alleviation of depressive symptoms and diminish psychological suffering. Furthermore, reducing PI/EA may not only alleviate but also prevent adolescent depression. In future studies, the simultaneous assessment of other resilience factors, such as social support, would help elucidate the nature of trait resilience and its effects. Such factors are likely to influence the connection between trait resilience and the change in depressive symptoms.

We conducted a supplementary analysis in different resilience change quintiles to explore the differences in the change in depressive symptoms. We noticed that the change in resilience was linearly associated with the change in depressive symptoms (Supplementary Material 1).

## Funding

This work was supported by the Academy of Finland, 352509.

## Ethics Statement

The study conformed to the standards set by the 7th revision of the Declaration of Helsinki (World Medical Association, 2013). The study protocol was approved by the Research Ethics Committee of Kuopio University Hospital.

## Consent

All participants provided written informed consent, and the consent was also approved by the caregivers of participants who were under 15 years old at the study baseline, as mandated by national guidelines. The parents of minors aged 15 or older were informed of the study.

## Conflicts of Interest

The authors declare no conflicts of interest.

## Clinical Trial Registration

The study protocol was approved by the Research Ethics Committee of Kuopio University Hospital.

## Supporting information


**Data S1:** Supporting information.

**Data S2:** Supporting information.

## Data Availability

Data available on request from the authors.

## References

[cpp70268-bib-0001] Alismail, A. M. , and M. O. Almulla . 2023. “Role of Psychological Resilience in Predicting Emotional Regulation Among Students of King Faisal University in Al‐Ahsa Governorate.” Eurasian Journal of Educational Research 105, no. 119: 139. 10.14689/ejer.2023.105.008.

[cpp70268-bib-0002] Askeland, K. G. , T. Bøe , K. Breivik , A. M. La Greca , B. Sivertsen , and M. Hysing . 2020. “Life Events and Adolescent Depressive Symptoms: Protective Factors Associated With Resilience.” PLoS ONE 15, no. 6: e0234109. 10.1371/journal.pone.0234109. 32502163 PMC7274383

[cpp70268-bib-0003] Beck, A. T. , R. A. Steer , R. Ball , and W. Ranieri . 1996. “Comparison of Beck Depression Inventories‐Ia and ‐Ii in Psychiatric Outpatients.” Journal of Personality Assessment 67, no. 3: 588–597. 10.1207/s15327752jpa6703_13. 8991972

[cpp70268-bib-0004] Berking, M. , A. Neacsiu , K. Comtois , and M. Linehan . 2009. “The Impact of Experiential Avoidance on the Reduction of Depression in Treatment for Borderline Personality Disorder.” Behaviour Research and Therapy 47, no. 8: 663–670. 10.1016/j.brat.2009.04.011. 19477434 PMC2771266

[cpp70268-bib-0006] Block, J. H. , and J. Block . 1980. “The Role of Ego‐Control and Ego‐Resiliency in the Organization of Behavior.” In Development of Cognition, Affect and Social Relations: The Minnesota Symposia on Child Psychology, edited by W. A. Collins , vol. 13, 39–101. Erlbaum.

[cpp70268-bib-0005] Block, J. , and A. M. Kraemen . 1996. “IQ and Ego‐Resiliency: Conceptual and Empirical Connections and Separateness.” Journal of Personality and Social Psychology 70, no. 2: 349–361. 8636887 10.1037//0022-3514.70.2.349

[cpp70268-bib-0007] Bond, F. W. , S. C. Hayes , R. A. Baer , et al. 2011. “Preliminary Psychometric Properties of the Acceptance and Action Questionnaire‐II: A Revised Measure of Psychological Inflexibility and Experiential Avoidance.” Behavior Therapy 42, no. 4: 676–688. 10.1016/j.beth.2011.03.007. 22035996

[cpp70268-bib-0008] Breslau, J. , M. Lane , N. Sampson , and R. C. Kessler . 2008. “Mental Disorders and Subsequent Educational Attainment in a US National Sample.” Journal of Psychiatric Research 42, no. 9: 708–716. 10.1016/j.jpsychires.2008.01.016. 18331741 PMC2748981

[cpp70268-bib-0009] Campbell‐Sills, L. , S. L. Cohan , and M. B. Stein . 2006. “Relationship of Resilience to Personality, Coping, and Psychiatric Symptoms in Young Adults.” Behavior Research and Therapy 44, no. 4: 585–599. 10.1016/j.brat.2005.05.001 15998508

[cpp70268-bib-0010] Campbell‐Sills, L. , and B. Stein M . 2007. “Psychometric Analysis and Refinement of the Connor–Davidson Resilience Scale (CD‐RISC): Validation of a 10‐Item Measure of Resilience.” Journal of Traumatic Stress 20, no. 6: 1019–1028. 10.1002/jts.20271. 18157881

[cpp70268-bib-0011] Chawla, N. , and B. Ostafin . 2007. “Experiential Avoidance as a Functional Dimensional Approach to Psychopathology: An Empirical Review.” Journal of Clinical Psychology 63, no. 9: 871–890. 10.1002/jclp.20400. 17674402

[cpp70268-bib-0012] Chen, Y.‐X. , S.‐H. Lin , P. S. Chen , et al. 2025. “Long‐Term Moderating Effect of Resilience Capacity on the Impact of Stressful Life Events on Depressive Symptoms After 6 Years in Outpatients With Depression and/or Anxiety.” Journal of Nervous and Mental Disease 213: 22–27. 10.1097/NMD.0000000000001808. 39787583

[cpp70268-bib-0013] Chung, J. , K. Lam , K. Y. Ho , et al. 2020. “Relationships Among Resilience, Self‐Esteem, and Depressive Symptoms in Chinese Adolescents.” Journal of Health Psychology 25, no. 13–14: 2396–2405. 10.1177/1359105318800159. 30229681

[cpp70268-bib-0102] Compas, B. E. , S. S. Jaser , A. H. Bettis , et al. 2017. “Coping, Emotion Regulation, and Psychopathology in Childhood and Adolescence: A Meta‐Analysis and Narrative Review.” Psychological Bulletin 143, no. 9: 939–991. 10.1037/bul0000110. 28616996 PMC7310319

[cpp70268-bib-0014] Connor, K. M. , and J. R. T. Davidson . 2003. “Development of a New Resilience Scale: The Connor–Davidson Resilience Scale (CD‐RISC).” Depression and Anxiety 18, no. 2: 76–82. 10.1002/da.10113. 12964174

[cpp70268-bib-0015] Cosco, T. D. , A. Kaushal , R. Hardy , M. Richards , D. Kuh , and M. Stafford . 2017. “Operationalising Resilience in Longitudinal Studies: A Systematic Review of Methodological Approaches.” Journal of Epidemiology & Community Health 71, no. 1: 98–104. 10.1136/jech-2015-206980. 27502781 PMC5256275

[cpp70268-bib-0017] Elliott, T. R. , Y. Y. Hsiao , N. A. Kimbrel , et al. 2015. “Resilience, Traumatic Brain Injury, Depression and Posttraumatic Stress Among Iraq/Afghanistan War Veterans.” Rehabilitation Psychology 60, no. 3: 263–276. 10.1037/rep0000050. 26214528 PMC5032656

[cpp70268-bib-0016] Elliott, T. R. , Y.‐Y. Hsiao , N. Kimbrel , et al. 2017. “Resilience and Traumatic Brain Injury Among Iraq/Afghanistan War Veterans: Differential Patterns of Adjustment and Quality of Life.” Journal of Clinical Psychology 73: 1160–1178. 10.1002/jclp.22414. 27922725 PMC5557690

[cpp70268-bib-0018] Elmore, A. L. , E. Crouch , and M. A. K. Chowdhury . 2020. “The Interaction of Adverse Childhood Experiences and Resiliency on the Outcome of Depression Among Children and Youth, 8–17 year olds.” Child Abuse Neglect 107: 104616. 10.1016/j.chiabu.2020.104616. 32645587 PMC7494539

[cpp70268-bib-0019] Fergus, S. , and M. A. Zimmerman . 2005. “Adolescent Resilience: A Framework for Understanding Healthy Development in the Face of Risk.” Annual Review of Public Health 26: 399–419. 10.1146/annurev.publhealth.26.021304.144357. 15760295

[cpp70268-bib-0020] First, M. , J. Williams , R. Karg , and R. Spitzer . 2017. Structured Clinical Interview for DSM‐5 (SCID‐5 for DSM‐5) Arlington. American Psychiatric Association.

[cpp70268-bib-0021] Garber, J. 2006. “Depression in Children and Adolescents Linking Risk Research and Prevention.” American Journal of Preventive Medicine 31, no. 6S1: S104–S125. 10.1016/j.amepre.2006.07.007. 17175406

[cpp70268-bib-0022] Gårdvik, K. , M. Rygg , T. Torgersen , J. Wallander , S. Lydersen , and M. Indredavik . 2021. “Association of Treatment Procedures and Resilience to Symptom Load Three‐Years Later in a Clinical Sample of Adolescent Psychiatric Patients.” BMC Psychiatry 21: 411. 10.1186/s12888-021-03417-6. 34412609 PMC8377856

[cpp70268-bib-0023] Goldenson, J. , I. Kitollari , and F. Lehman . 2021. “The Relationship Between ACE:s, Trauma‐Related Psychopathology and Resilience in Vulnerable Youth: Implications for Screening and Treatment.” Journal of Child & Adolescent Trauma 14: 151–160. 10.1007/s40653-020-00308-y. 33708289 PMC7900283

[cpp70268-bib-0104] Hartung, C. , A. Lerer , Y. Anokwa , C. Tseng , and G. Borriello . 2010. “Open Data Kit: Tools to Build Information Services for Developing Regions.” In *Proceedings of the 4th ACM/IEEE International Conference on Information and Communication Technologies and Development*, 1–12. 10.1145/2369220.2369236.

[cpp70268-bib-0106] Hayes, A. F. 2018. Introduction to Mediation, Moderation, and Conditional Process Analysis: A Regression‐Based Approach. 2nd ed. Guilford Press.

[cpp70268-bib-0108] Hayes, L. , C. P. Boyd , and J. Sewell . 2011. “Acceptance and Commitment Therapy for the Treatment of Adolescent Depression: A Pilot Study in a Psychiatric Outpatient Setting.” Mindfulness 2: 86–94.

[cpp70268-bib-0024] Hayes, S. C. , J. B. Luoma , F. W. Bond , A. Masuda , and J. Lillis . 2006. “Acceptance and commitment therapy: Model, processes, and outcomes.” Behavior Research and Therapy 44, no. 1: 1–25. 10.1016/j.brat.2005.06.006. 16300724

[cpp70268-bib-0103] Hayes, S. C. , K. G. Wilson , E. V. Gifford , and V. Follete . 1996. “Experiential Avoidance and Behavioral Disorders: A Functional Dimensional Approach to Diagnosis and Treatment.” Journal of Consulting and Clinical Psychology 64, no. 6: 1152–1168. 10.1037/0022-006X.64.6.1152. 8991302

[cpp70268-bib-0026] Hjemdal, O. , T. Aune , T. Reinfjell , and T. C. Stiles . 2007. “Resilience as a Predictor of Depressive Symptoms: A Correlational Study With Young Adolescents.” Clinical Child Psychology and Psychiatry 12, no. 1: 91–104. 10.1177/1359104507071062. 17375811

[cpp70268-bib-0027] Hjemdal, O. , P. A. Vogel , S. Solem , K. Hagen , and T. C. Stiles . 2011. “The Relationship Between Resilience and Levels of Anxiety, Depression, and Obsessive‐Compulsive Symptoms in Adolescents.” Clinical Psychology & Psychotherapy 18, no. 4: 314–321. 10.1002/cpp.719. 20806419

[cpp70268-bib-0028] Hu, T. , D. Zhang , and J. Wang . 2015. “A Meta‐Analysis of Trait Resilience and Mental Health.” Personality and Individual Differences 76: 18–27. 10.1016/j.paid.2014.11.039.

[cpp70268-bib-0029] Kalisch, R. , D. G. Baker , U. Basten , et al. 2017. “The Resilience Framework as a Strategy to Combat Stress‐Related Disorders.” Nature Human Behaviour 1: 784–790. 10.1038/s41562-017-0200-8. 31024125

[cpp70268-bib-0031] Kalisch, R. , M. Muller , and O. Tuscher . 2015. “A Conceptual Framework for the Neurobiological Study of Resilience.” Behavioral and Brain Sciences 38: 1–79. 10.1017/S0140525X1400082X. 25158686

[cpp70268-bib-0033] Keulen, J. , D. Matthijssen , J. Schraven , M. Deković , and D. Bodden . 2023. “The Effectiveness and Cost‐Effectiveness of Acceptance and Commitment Therapy as a Transdiagnostic Intervention for Transitional‐Age Youth: Study Protocol of a Randomized Controlled Trial.” BMC Psychiatry 23: 51. 10.1186/s12888-023-04535-z. 36658510 PMC9850708

[cpp70268-bib-0100] Konaszewski, K. , M. Niesiobędzka , and J. Surzykiewicz . 2021. “Resilience and Mental Health Among Juveniles: Role of Strategies for Coping with Stress.” Health Quality Life Outcomes 19: 58. 10.1186/s12955-021-01701-3. PMC7891003 33602278

[cpp70268-bib-0034] Konradt, C. E. , T. D. Cardoso , T. C. Mondin , et al. 2018. “Impact of Resilience on the Improvement of Depressive Symptoms After Cognitive Therapies for Depression in a Sample of Young Adults.” Trends in Psychiatry and Psychotherapy 40: 226–231. 10.1590/2237-6089-2017-0047. 30304118

[cpp70268-bib-0035] Lazarus, R. S. 1984. Stress, Appraisal, and Coping, 464. Springer.

[cpp70268-bib-0036] Levin, M. E. , C. McLane , S. Daflos , et al. 2014. “Examining Psychological Inflexibility as a Transdiagnostic Process Across Psychological Disorders.” Journal of Contextual Behavioral Science 3, no. 3: 155–163. 10.1016/j.jcbs.2014.06.003. 29057212 PMC5650239

[cpp70268-bib-0037] Li, Y. , and P. Zheng . 2025. “Trait Resilience Protects Against Social Anxiety in College Students Through Emotion Regulation and Coping Strategies.” Scientific Reports 15: 28143. 10.1038/s41598-025-13674-0. 40751070 PMC12317036

[cpp70268-bib-0038] Ma, A. , Y. Yang , S. Guo , X. Li , S. Zhang , and H. Chang . 2022. “The Impact of Adolescent Resilience on Mobile Phone Addiction During COVID‐19 Normalization and Flooding in China: A Chain Mediating.” Frontiers in Psychology 13: 865306. 10.3389/fpsyg.2022.865306. 35814111 PMC9261930

[cpp70268-bib-0039] Masten, A. S. , C. M. Lucke , K. M. Nelson , and I. C. Stallworthy . 2021. “Resilience in Development and Psychopathology: Multisystem Perspectives.” Annual Review of Clinical Psychology 17: 521–549. 10.1146/annurev-clinpsy-081219-120307. 33534615

[cpp70268-bib-0040] McGrath, J. J. , A. Al‐Hamzawi , J. Alonso , et al. 2023. “Age of Onset and Cumulative Risk of Mental Disorders: A Cross‐National Analysis of Population Surveys From 29 Countries.” Lancet Psychiatry 10, no. 9: 668–681. 37531964 10.1016/S2215-0366(23)00193-1 PMC10529120

[cpp70268-bib-0041] McLaughlin, K. A. , N. L. Colich , A. M. Rodman , and D. G. Weissman . 2020. “Mechanisms Linking Childhood Trauma Exposure and Psychopathology: A Transdiagnostic Model of Risk and Resilience.” BMC Medicine 18: 96. 10.1186/s12916-020-01561-6. 32238167 PMC7110745

[cpp70268-bib-0042] Mellick, W. H. , J. A. Mills , E. B. Kroska , C. A. Calarge , L. Sharp , and L. N. Dindo . 2019. “Experiential Avoidance Predicts Persistence of Major Depressive Disorder and Generalized Anxiety Disorder in Late Adolescence.” Journal of Clinical Psychiatry 80, no. 6: 18m12265. 10.4088/JCP.18m12265. PMC6854672 31644841

[cpp70268-bib-0043] Mellick, W. H. , S. Vanwoerden , and C. Sharp . 2017. “Experiential Avoidance in the Vulnerability to Depression Among Adolescent Females.” Journal of Affective Disorders 208: 497–502. 10.1016/j.jad.2016.10.034. 27814961

[cpp70268-bib-0044] Mervin, R. M. , C. A. Timko , A. A. Moskovich , K. Konrad Ingle , M. C. Bulik , and N. L. Zucker . 2010. “Psychological Inflexibility and Symptom Expression in Anorexia Nervosa.” Eating Disorders 19, no. 1: 62–82. 10.1080/10640266.2011.533606. 21181580

[cpp70268-bib-0045] Mesman, E. , A. Vreeker , and M. Hillergers . 2021. “Resilience and Mental Health in Children and Adolescents: An Update of the Recent Literature and Future Directions.” Current Opinion in Psychiatry 34: 586–592. 10.1097/YCO.0000000000000741. 34433193 PMC8500371

[cpp70268-bib-0046] Meule, A. , K. Lieb , A. Chmitorz , and U. Voderholzer . 2024. “Resilience and Depressive Symptoms in Inpatients With Depression: A Cross‐Lagged Panel Model.” Clinical Psychology & Psychotherapy 31, no. 1: e2926. 10.1002/cpp.292. 37885282

[cpp70268-bib-0047] Meyer, E. C. , A. Kotte , N. A. Kimbrel , et al. 2019. “Predictors of Lower‐Than‐Expected Posttraumatic Symptom Severity in War Veterans: The Influence of Personality, Self‐Reported Trait Resilience, and Psychological Flexibility.” Behaviour Research and Therapy 113: 1–8. 10.1016/j.brat.2018.12.005. 30553859

[cpp70268-bib-0048] Miller, A. M. , and P. J. Chandler . 2002. “Acculturation, Resilience, and Depression in Midlife Women From the Former Soviet Union.” Nursing Research 51: 26–32. 10.1097/00006199-200201000-00005. 11822566

[cpp70268-bib-0049] Min, J. A. , Y. E. Jung , D. J. Kim , et al. 2013. “Characteristics Associated With Low Resilience in Patients With Depression and/or Anxiety Disorders.” Quality of Life Research 22: 231–241. 10.1007/s11136-012-0153-3. 22485024

[cpp70268-bib-0050] Min, J. A. , N. B. Lee , C. U. Lee , C. Lee , and J. H. Chae . 2012. “Low Trait Anxiety, High Resilience, and Their Interaction as Possible Predictors for Treatment Response in Patients With Depression.” Journal of Affective Disorders 137, no. 1–3: 61–69. 10.1016/j.jad.2011.12.026. 22244377

[cpp70268-bib-0051] Mondolin, V. , H. Karlsson , L. Perasto , J. J. Tuulari , L. Karlsson , and E.‐L. Kataja . 2024. “Understanding Resilience in Parents: Longitudinal Examination of Trait Resilience, Stressful Life Events, and Psychological Distress Symptoms—Insights From the FinnBrain Study.” Stress and Health 40: e3516. 10.1002/smi.3516. 39620277 PMC11636431

[cpp70268-bib-0052] Nakajima, S. , Y. Kaneko , N. Fujii , et al. 2023. “Transdiagnostic Association Between Subjective Insomnia and Depressive Symptoms in Major Psychiatric Disorders.” Frontiers in Psychiatry 14: 1114945. 10.3389/fpsyt.2023.1114945. 37168089 PMC10165079

[cpp70268-bib-0053] Nrugham, L. , A. Holen , and A. M. Sund . 2010. “Associations Between Attempted Suicide, Violent Life Events, Depressive Symptoms, and Resilience in Adolescents and Young Adults.” Journal of Nervous and Mental Disease 198: 131–136. 10.1097/NMD.0b013e3181cc43a2. 20145488

[cpp70268-bib-0054] Ong, A. D. , C. S. Bergeman , and S. M. Boker . 2009. “Resilience Comes of Age: Defining Features in Later Adulthood.” Journal of Personality 77, no. 6: 1777–1804. 10.1111/j.1467-6494.2009.00600.x. 19807864 PMC2807734

[cpp70268-bib-0055] Pardeller, S. , G. Kemmler , C. M. Hoertnagl , and A. Hofer . 2020. “Associations Between Resilience and Quality of Life in Patients Experiencing a Depressive Episode.” Psychiatric Research 292: 113353. 10.1016/j.psychres.2020.113353. 32771836

[cpp70268-bib-0056] Poole, J. C. , K. A. Dobson , and D. Pusch . 2017. “Childhood Adversity and Adult Depression: The Protective Role of Psychological Resilience.” Child Abuse & Neglect 64: 89–100. 10.1016/j.chiabu.2016.12.012. 28056359

[cpp70268-bib-0057] Rutter, M. 2006. “Implications of Resilience Concepts for Scientific Understanding.” Annals. New York Academy of Sciences 1094: 1–12. 10.1196/annals.1376.002. 17347337

[cpp70268-bib-0058] San Román‐Mata, S. , P. Puertas‐Molero , J. L. Ubago‐Jiménez , and G. González‐Valero . 2020. “Benefits of Physical Activity and Its Associations With Resilience, Emotional Intelligence, and Psychological Distress in University Students From Southern Spain.” International Journal of Environmental Research and Public Health 17. 10.3390/ijerph17124474. PMC7344387 32580322

[cpp70268-bib-0059] Schafer, J. O. , E. Naumann , E. A. Holmes , B. Tuschen‐Caffier , and A. C. Samson . 2017. “Emotion Regulation Strategies in Depressive and Anxiety Symptoms in Youth: A Meta‐Analytic Review.” Journal of Youth and Adolescence 46, no. 2: 261–276. 10.1007/s10964-016-0585-0. 27734198

[cpp70268-bib-0060] Schei, J. , T. S. Nøvik , P. H. Thomsen , S. Lydersen , M. S. Indredavik , and T. Jozefiak . 2018. “What Predicts a Good Adolescent to Adult Transition in ADHD? The Role of Self‐Reported Resilience.” Journal of Attention Disorders 22, no. 6: 547–560. 10.1177/1087054715604362. 26399710

[cpp70268-bib-0061] Seok, J. H. , K. U. Lee , W. Kim , et al. 2012. “Impact of Early‐Life Stress and Resilience on Patients With Major Depressive Disorder.” Yonsei Medical Journal 53, no. 6: 1093–1098. 10.3349/ymj.2012.53.6.1093. 23074107 PMC3481369

[cpp70268-bib-0062] Shah, J. L. , J. Scott , P. D. McGorry , et al. 2020. “Transdiagnostic Clinical Staging in Youth Mental Health: A First International Consensus Statement.” World Psychiatry: Official Journal of the World Psychiatric Association (WPA) 19, no. 2: 233–242. 10.1002/wps.20745. 32394576 PMC7215079

[cpp70268-bib-0063] Shapero, B. G. , A. Farabaugh , O. Terechina , et al. 2019. “Understanding the Effects of Emotional Reactivity on Depression and Suicidal Thoughts and Behaviors: Moderating Effects of Childhood Adversity and Resilience.” Journal of Affective Disorders 245: 419–427. 10.1016/j.jad.2018.11.033. 30423470

[cpp70268-bib-0064] Siu, A. M. H. , and D. T. L. Shek . 2010. “Social Problem Solving as a Predictor of Well‐Being in Adolescents and Young Adults.” Social Indicators Research 95, no. 3: 393–406. 10.1007/s11205-009-9527-5.

[cpp70268-bib-0065] Smith, B. W. , J. Dalen , K. Wiggins , E. Tooley , P. Christopher , and J. Bernard . 2008. “The Brief Resilience Scale: Assessing the Ability to Bounce Back.” International Journal of Behavioral Medicine 15: 194–200. 10.1080/10705500802222972. 18696313

[cpp70268-bib-0066] Stainton, A. , K. Chisholm , N. Kaiser , et al. 2018. “Resilience as a Multimodal Dynamic Process.” Early Intervention in Psychiatry 13: 725–732. 10.1111/eip.12726. 30126047

[cpp70268-bib-0067] Steer, R. A. , D. A. Clark , A. T. Beck , and W. F. Ranieri . 1998. “Common and Specific Dimensions of Self‐Reported Anxiety and Depression: The BDI‐II versus the BDI‐IA.” Behaviour Research and Therapy 37: 183–190. 10.1016/s0005-7967(98)00087-4. 9990749

[cpp70268-bib-0068] Thapar, A. , S. Collishaw , DPhil , D. S. Pine , and A. K. Thapar . 2012. “Depression in Adolescence.” Lancet 379, no. 9820: 1056–1067. 10.1016/S0140-6736(11)60871-4. 22305766 PMC3488279

[cpp70268-bib-0069] Ungar, M. , and L. Theron . 2020. “Resilience and Mental Health: How Multisystemic Processes Contribute to Positive Outcomes.” Lancet Psychiatry 7, no. 5: 441–448. 10.1016/S2215-0366(19)-30434-1. 31806473

[cpp70268-bib-0070] Venta, A. , C. Sharp , and J. Hart . 2012. “The Relation Between Anxiety Disorder and Experiential Avoidance in Inpatient Adolescents.” Psychological Assessment 24, no. 1: 240–248. 10.1037/a0025362. 21895380

[cpp70268-bib-0071] Walser, R. D. , D. W. Garvert , B. E. Karlin , M. Trockel , D. M. Ryu , and C. B. Taylor . 2015. “Effectiveness of Acceptance and Commitment Therapy in Treating Depression and Suicidal Ideation in Veterans.” Behaviour Research and Therapy 74: 25–31. 10.1016/j.brat.2015.08.012. 26378720

[cpp70268-bib-0072] Wang, Y. , J. Tian , and Q. Yang . 2024. “Experiential Avoidance Process Model: A Review of the Mechanism for the Generation and Maintenance of Avoidance Behavior.” Psychiatry and Clinical Psychopharmacology 34, no. 2: 179–190. 39165887 10.5152/pcp.2024.23777 PMC11332439

[cpp70268-bib-0073] Waugh, C. E. , and E. H. W. Koster . 2015. “A Resilience Framework for Promoting Stable Remission From Depression.” Clinical Psychology Review 41: 49–60. 10.1016/j.cpr.2014.05.004. 24930712

[cpp70268-bib-0074] Whiteford, H. A. , L. Degenhardt , J. Rehm , et al. 2013. “Global Burden of Disease Attributable to Mental and Substance Use Disorders: Findings From the Global Burden of Disease Study 2010.” Lancet 382, no. 9904: 1575–1586.nn6. 23993280 10.1016/S0140-6736(13)61611-6

[cpp70268-bib-0105] World Medical Association . 2013. “World Medical Association Declaration of Helsinki: Ethical Principles for Medical Research Involving Human Subjects.” JAMA 310, no. 20: 2191–2194. 10.1001/jama.2013.281053. 24141714

[cpp70268-bib-0075] Wu, Y. , Z. Sang , X. Zhang , and J. Margraf . 2020. “The Relationship Between Resilience and Mental Health in Chinese College Students: A Longitudinal Cross‐Lagged Analysis.” Frontiers in Psychology 11: 108. 10.3389/fpsyg.2020.00108. 32116918 PMC7012791

[cpp70268-bib-0107] Zhang, C. , M. Ye , Y. Fu , et al. 2020. “The Psychological Impact of the COVID‐19 Pandemic on Teenagers in China.” Journal of Adolescent Health 67: 747–755. 10.1016/j.jadohealth.2020.08.026. PMC7543885 33041204

