# Supplementary figures and images for: The Change in Trait Resilience Predicts the Alleviation of Transdiagnostic Depressive Symptoms in Outpatient Adolescents: The Mediating Role of the Change in Psychological Inflexibility/Experiential Avoidance
Source: Clin Psychol Psychother. 2026 Apr 9;33(2):e70268. doi: 10.1002/cpp.70268 (PMC13063102; doi:10.1002/cpp.70268)

Supplementary material 1


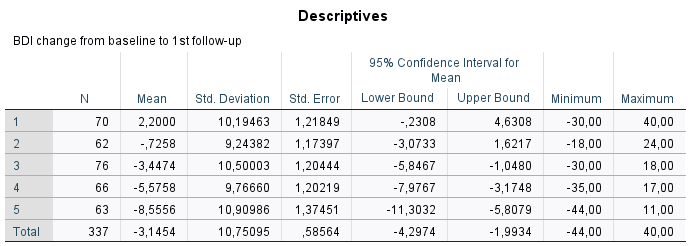


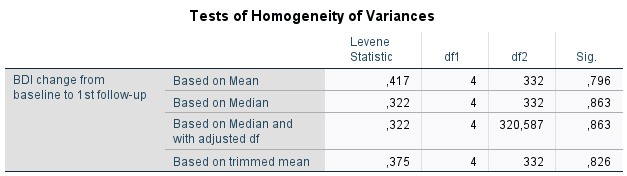


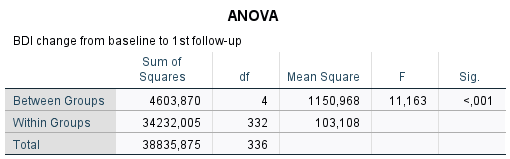


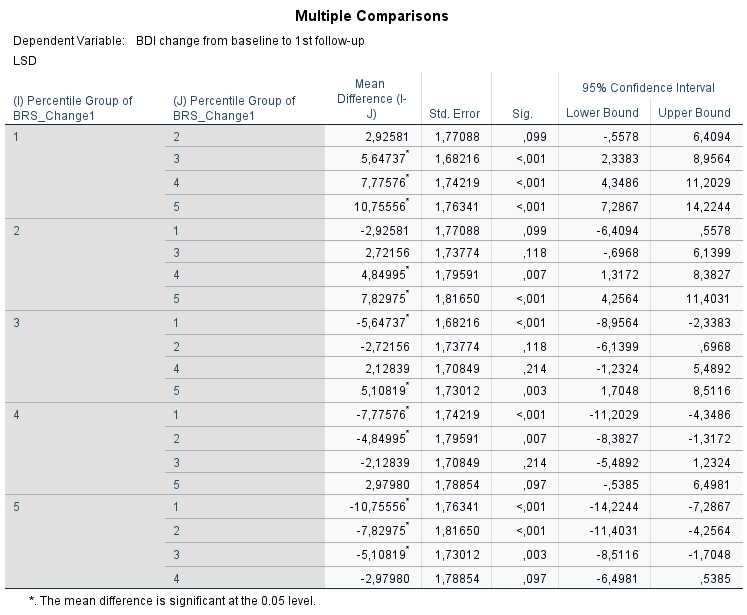


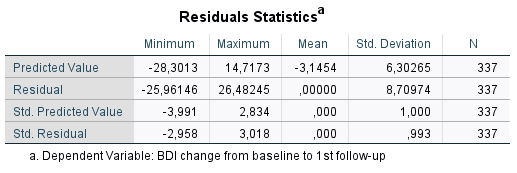


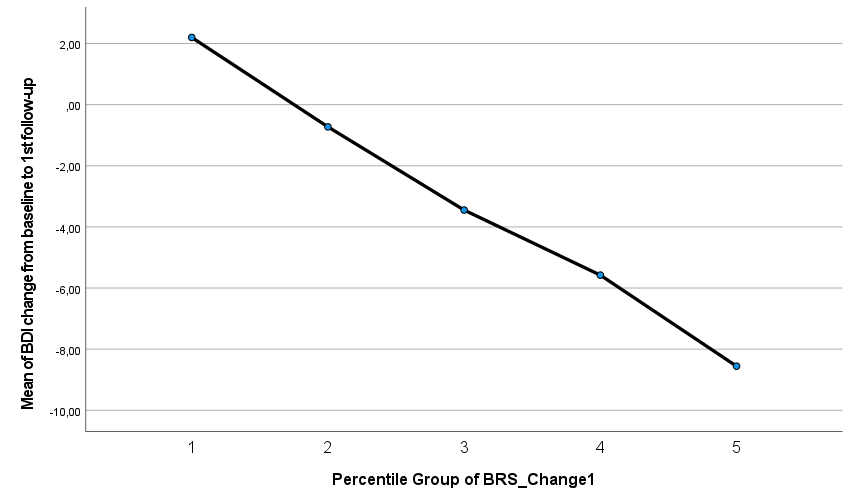


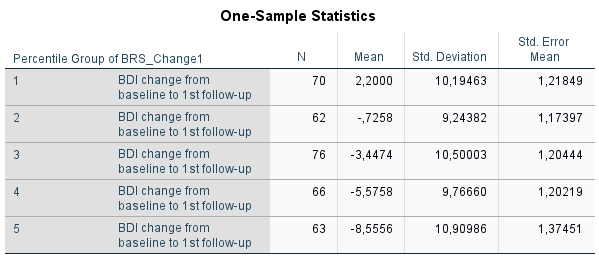


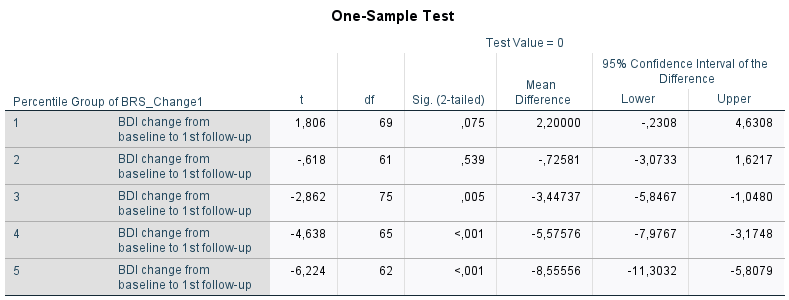


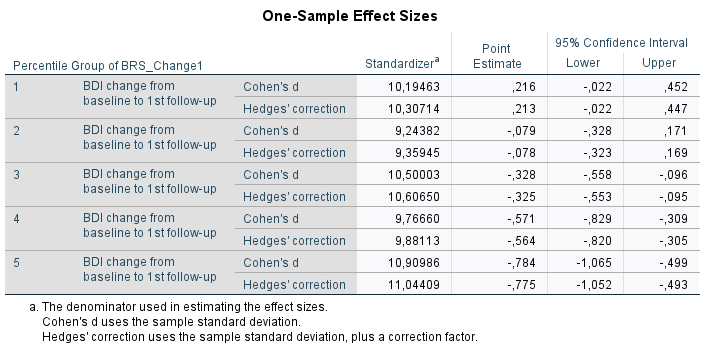


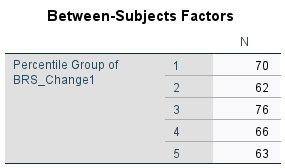


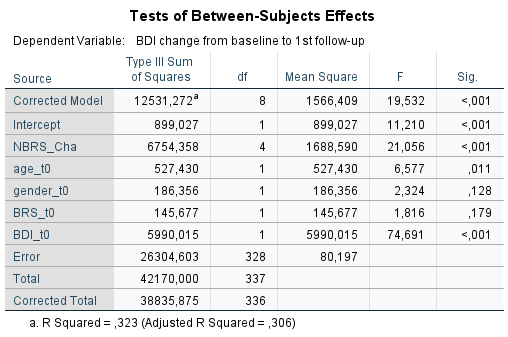


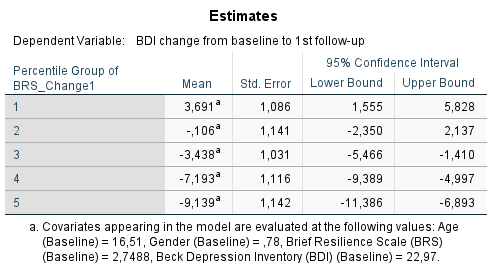


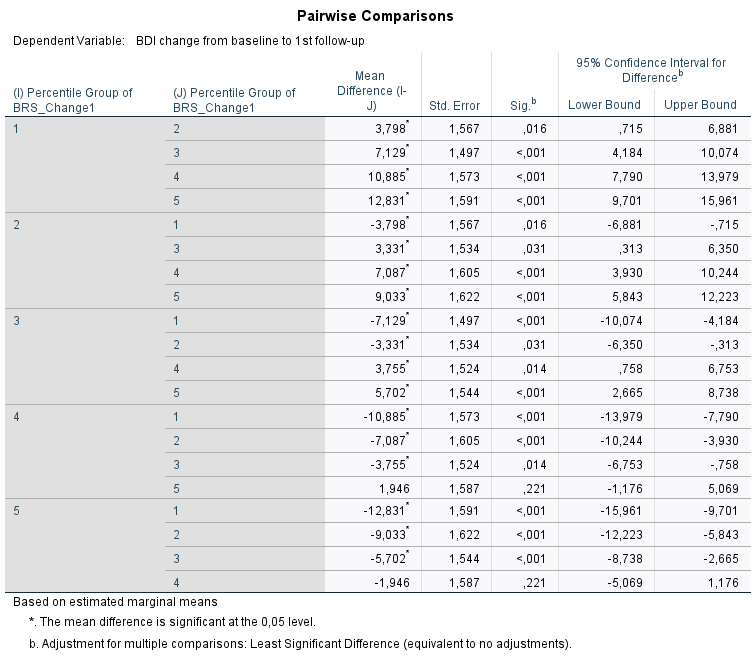


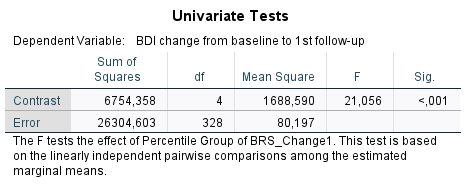

Supplement: Supplementary file 1 — Data S1: Supporting information. [file CPP-33-e70268-s001.docx]
