# Supplementary material for: The Change in Trait Resilience Predicts the Alleviation of Transdiagnostic Depressive Symptoms in Outpatient Adolescents: The Mediating Role of the Change in Psychological Inflexibility/Experiential Avoidance
Source: Clin Psychol Psychother. 2026 Apr 9;33(2):e70268. doi: 10.1002/cpp.70268 (PMC13063102; doi:10.1002/cpp.70268)

1. Linearity between the change in trait resilience and the change in depressive symptoms


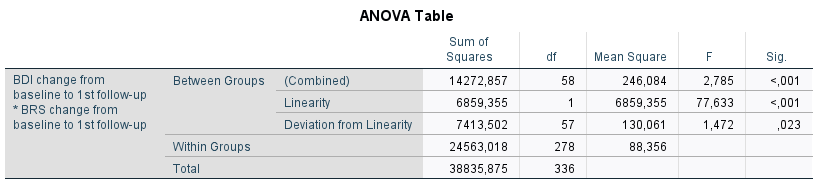


Linearity between the change in trait resilience and the change in PI/EA


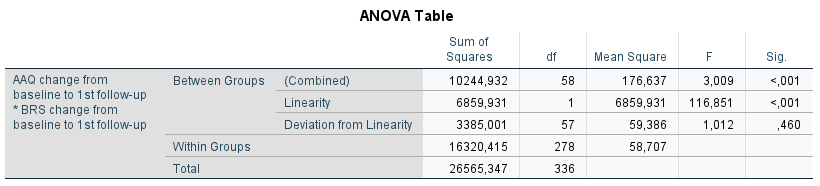


Linearity between the change in PI/EA and the change in depressive symptoms


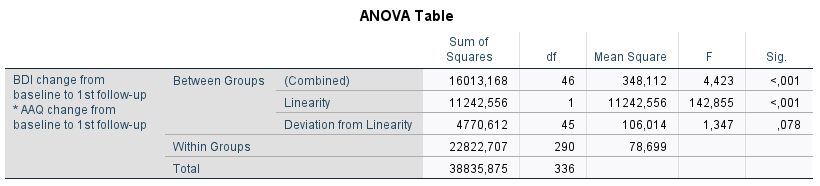


2.)Multicollinearity


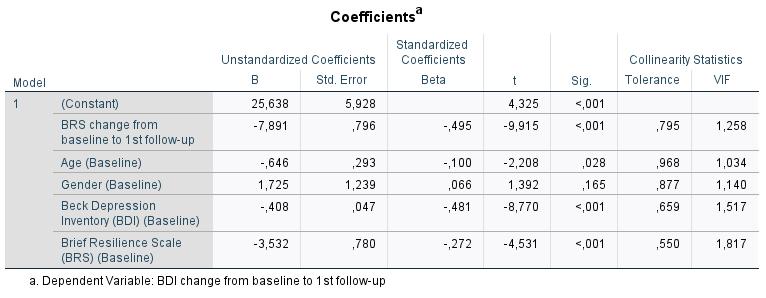


1. Homoscedasticity


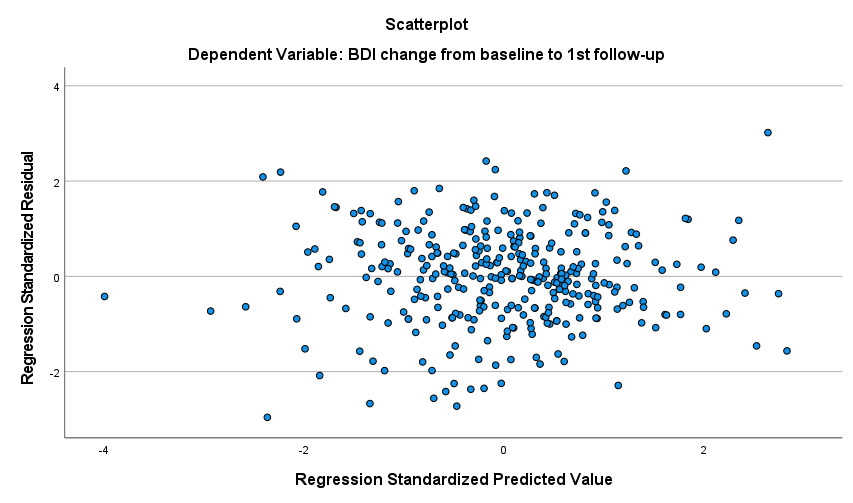


1. Outliers


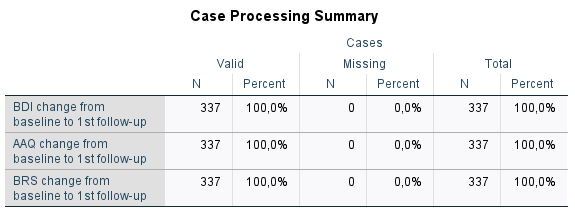


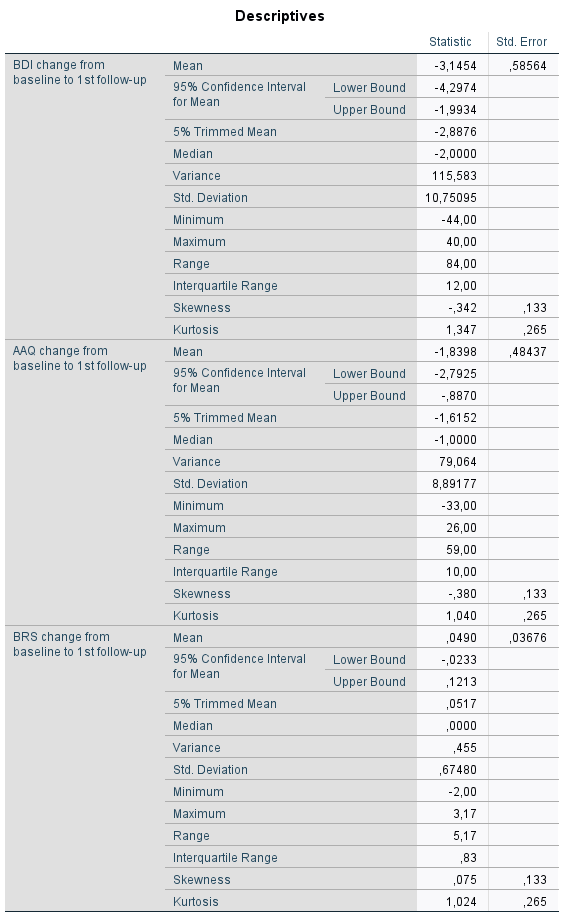


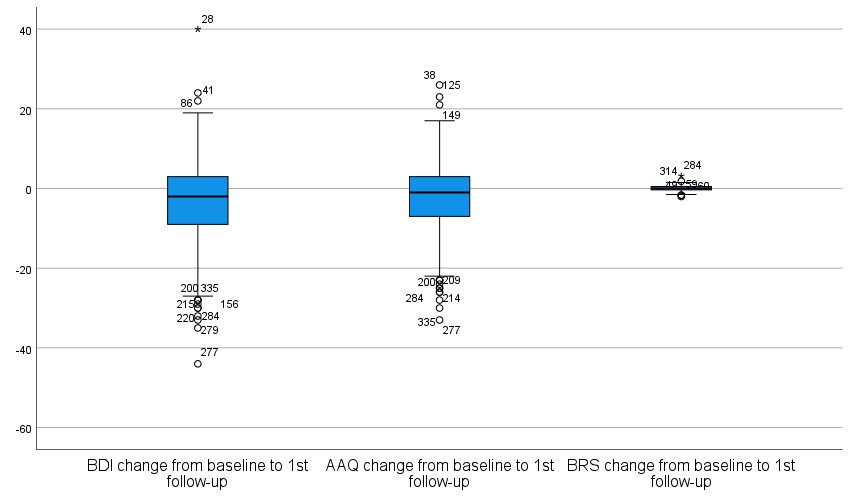

Supplement: Supplementary file 2 — Data S2: Supporting information. [file CPP-33-e70268-s002.docx]
